# QSAR Study of Antimicrobial 3-Hydroxypyridine-4-one and 3-Hydroxypyran-4-one Derivatives Using Different Chemometric Tools

**DOI:** 10.3390/ijms9122407

**Published:** 2008-12-02

**Authors:** Razieh Sabet, Afshin Fassihi

**Affiliations:** Department of Medicinal Chemistry, Faculty of Pharmacy, Isfahan University of Medical Sciences, 81746-73461, Isfahan, Iran. E-Mail: Sabet@pharm.mui.ac.ir

**Keywords:** 3-Hydroxypyridine-4-one, 3-hydroxypyran-4-one, QSAR, Chemometrics

## Abstract

A series of 3-hydroxypyridine-4-one and 3-hydroxypyran-4-one derivatives were subjected to quantitative structure-antimicrobial activity relationships (QSAR) analysis. A collection of chemometrics methods, including factor analysis-based multiple linear regression (FA-MLR), principal component regression (PCR) and partial least squares combined with genetic algorithm for variable selection (GA-PLS) were employed to make connections between structural parameters and antimicrobial activity. The results revealed the significant role of topological parameters in the antimicrobial activity of the studied compounds against *S. aureus* and *C. albicans*. The most significant QSAR model, obtained by GA-PLS, could explain and predict 96% and 91% of variances in the pIC_50_ data (compounds tested against *S. aureus*) and predict 91% and 87% of variances in the pIC_50_ data (compounds tested against *C. albicans*), respectively.

## 1. Introduction

Quantitative structure activity relationships (QSAR) studies, as one of the most important areas in chemometrics, give information that is useful for molecular design and medicinal chemistry [1–5]. QSAR models are mathematical equations constructing a relationship between chemical structures and biological activities. These models have another ability, which is providing a deeper knowledge about the mechanism of biological activity. In the first step of a typical QSAR study one needs to find a set of molecular descriptors with the higher impact on the biological activity of interest [6–9]. A wide range of descriptors has been used in QSAR modeling. These descriptors have been classified into different categories, including constitutional, geometrical, topological, quantum chemical and so on. There are several variable selection methods including multiple linear regression (MLR), genetic algorithm (GA), partial least squares (PLS), principle component or factor analysis (PCA/FA), and so on. [7–9]. MLR yields models that are simpler and easier to interpret than PCR and PLS, because these methods perform regression on latent variables that don’t have physical meaning. Due to the co-linearity problem in MLR analysis, one may remove the collinear descriptors before MLR model development. MLR equations can describe the structure activity relationships well but some information will be discarded in MLR analysis. On the other hand, factor analysis–based methods such as PLS regression can handle the collinear descriptors and therefore better predictive models will be obtained by PLS method [10].

It is almost 120 years since physicians revealed that the coincidence of blood and bacteria in a wound may cause a life-threatening infection. It has also been shown that blood or hemoglobin enhance the lethality of intraperitoneal or subcutaneous inocula of bacteria such as *Escherichia coli*. The effective component of hemoglobin is iron, and various soluble iron compounds exert an equivalent effect [11]. Administration of iron compounds to the host can increase the virulence of *Escherichia coli*, *Listeria monocytogenes*, *Salmonella typhimurium* and other pathogens [12]. In fact, iron is an essential element required for the growth and virulence of virtually all microbial pathogens [13, 14]. The availability of iron is critically important in host-parasite interactions [15]. Vertebrate hosts withhold iron from microbial invaders as a major defence mechanism against infection [13, 15]. This task is achieved by sequestration of iron with iron-binding proteins, the most abundant, haemoproteins [16]. Some natural antibiotics, called siderophores, are low-molecular-weight chelating agents that form stable complexes with iron [17, 18]. There are many reports of the antimicrobial activity of chelating agents with different chemical structures [19–22]. Kojic acid (5-hydroxy-2-hydroxymethyl-pyran-4-one) and its 3-hydroxypyranones derivatives are examples of these compounds [19]. The bidentate chelating ligand 3-hydroxypyranone, which has a catechol-like function, forms stable complexes with several metal ions such as Fe^3+^. *In vitro* antibacterial and antifungal activities of 3-hydroxy-pyridinones, bioisoster derivatives of 3-hydroxypyranones with metal chelating ability have been described. They have an inhibitory effect on the growth of *Escherichia coli, Listeria inocua* and *Staphylococcus aureus* [22]. More recently antibacterial and antifungal activities of carboxamide derivatives of 3-hydroxypyranones, 5-hydroxypyranones and 5-hydroxypyridinones have been reported [23, 24].

Few reports of antimicrobial studies of 3-hydroxypyridine-4-one and 3-hydroxypyran-4-one derivatives are available [19, 21–25] and in those they were not the subject of QSAR studies. Preliminary QSAR models for a series of such derivatives have been investigated by Fassihi *et al.* [25]. The antimicrobial activity against *C. albicans*, *S. aureus* and *P. aeroginosa* was the subject of MLR analysis in this preliminary study. MLR models revealed the best relationship between the antimicrobial activity and structural properties against *S. aureus* and *C. albicans*. In the present paper, more than 600 topological, geometrical, constitutional, functional group, electrostatic, quantum and chemical descriptors were used, for the development of QSAR equations, different methods were applied for the antimicrobial activity of the studied compounds against *S. aureus* and *C. albicans*. These methods where: (i) genetic algorithm - partial least squares (GA-PLS), (ii) MLR with factor analysis as the data pre-processing step for variable selection (FA-MLR) and (iii) principal component regression analysis (PCRA). The correlation coefficient (*r*), standard error of regression (*SE*), r^2^cv (Q^2^) and RMScv (STD(r)) were employed to judge the validity of regression equation.

## 2. Experimental Section

### 2.1. Software

The two-dimensional structures of molecules were drawn using the Hyperchem 7.0 software. The final geometries were obtained with the semi-empirical AM1 method in the Hyperchem program. The molecular structures were optimized using the Polak-Ribiere algorithm until the root mean square gradient was 0.01 kcal mol^−1^. The resulted geometry was transferred into Dragon program package, which was developed by Milano Chemometrics and QSAR Group [26]. The z-matrix of the structures was provided by the software and transferred to the Gaussian 98 program. Complete geometry optimization was performed taking the most extended conformation as starting geometries. Semi-empirical molecular orbital calculation (AM1) of the structures was preformed using Gaussian 98 program [27]. MATLAB software (version 7.1 Math Work Inc.) was used for the PLS regression method.

### 2.2. Data set and descriptor generation

The biological data used in this study are antimicrobial activity, (in terms of –log MIC), of a set of 3-hydroxypyridine-4-one and 3-hydroxypyran-4-one derivatives [23, 24, 25]. The structural features of these compounds are listed in Table 1 and then used for subsequent QSAR analysis as dependent variables. The large number of molecular descriptors was calculated using Hyperchem, Dragon package and Gaussian 98. Some chemical parameters including molecular volume (V), molecular surface area (SA), hydrophobicity (LogP), hydration energy (HE) and molecular polarizability (MP) were calculated using Hyperchem Software. Dragon software calculated different functional groups, topological, geometrical and constitutional descriptors for each molecule.

Gaussian 98 was employed for calculation of different quantum chemical descriptors including, dipole moment (DM), local charges, HOMO and LOMO energies. Hardness (η), softness (S), electronegativity (χ) and electrophilicity (ω) were calculated according to the method proposed by Thanikaivelan *et al*. [28].

Constitutional, topological, geometrical, functional group, quantum and physicochemical indices were used in this study; brief description of some of them is listed in Table 2.

### 2.3. Data screening and model building

The calculated descriptors were collected in a data matrix whose number of rows and columns were the number of molecules and descriptors, respectively. Genetic algorithm - partial least squares (GA-PLS), MLR with factor analysis as the data pre-processing step for variable selection (FA-MLR) and principal component regression analysis (PCRA) methods were used to derive the QSAR equations and feature selection was performed by the use of genetic algorithm (GA). The genetic algorithms are efficient methods for function minimization. In descriptor selection context, the prediction error of the model built upon a set of features is optimized [29].

In this study, to model the structure-antimicrobial activity relationships better, genetic algorithm-partial least square (GA-PLS) was employed [30, 31]. Partial least squares (PLS) linear regression is a recent technique that generalizes and combines features from principal component analysis and multiple regressions. PLS is a method suitable for overcoming the problems in MLR related to multicollinear or over-abundant descriptors [10].

Application of PLS method thus allows the construction of larger QSAR equations while still avoiding over-fitting and eliminating most variables. This method is normally used in combination with cross-validation to obtain the optimum number of components [32, 33]. The PLS regression method used was the NIPALS-based algorithm existed in the chemometrics toolbox of MATLAB software (version 7.1 Math Work Inc.). In order to obtain the optimum number of factors based on the Haaland and Thomas F-ratio criterion, leave-one-out cross-validation procedure was used [34].

In our previous study the classical approach of multiple regression technique was used for developing QSAR relation [25]. Here, FA-MLR was also performed on the dataset. Factor analysis (FA) was used to reduce the number of variables and to detect structure in the relationships between them. This data-processing step is applied to identify the important predictor variables and to avoid collinearities among them [35]. Principle component regression analysis, PCRA, was also tried for the dataset along with FA-MLR. With PCRA collinearities among **X** variables are not a disturbing factor and the number of variables included in the analysis may exceed the number of observations [36]. In this method, factor scores, as obtained from FA, are used as the predictor variables [35]. In PCRA, all descriptors are assumed to be important while the aim of factor analysis is to identify relevant descriptors.

## 3. Results and Discussion

### 3.1. GA-PLS

**Figure 1.**
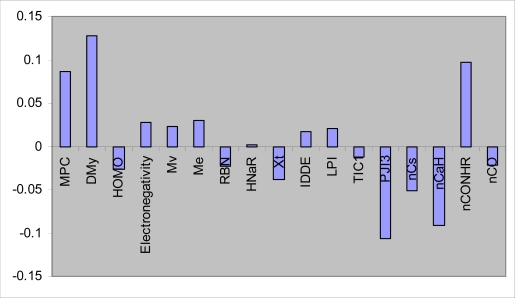
PLS regression coefficients for the variables used in GA-PLS model (against *S. aureus*).

In PLS analysis, the descriptors data matrix is decomposed to orthogonal matrices with an inner relationship between the dependent and independent variables. Therefore, unlike MLR analysis, the multicolinearity problem in the descriptors is omitted by PLS analysis. Because a minimal number of latent variables are used for modeling in PLS; this modeling method coincides with noisy data better than MLR. In order to find the more convenient set of descriptors in PLS modeling, genetic algorithm was used. To do so, many different GA-PLS runs were conducted using different initial set of populations. The data set (compounds tested against *S. aureus*, n = 31) was divided into two groups: calibration set (n = 25) and prediction set (n = 6). Given 25 calibration samples; the leave-one-out cross-validation procedure was used to find the optimum number of latent variables for each PLS model. The most convenient GA-PLS model that resulted in the best fitness contained 17 indices, 5 of them being those obtained by MLR. The PLS estimate of coefficients for these descriptors are given in Figure 1.

As it is observed, a combination of quantum, topological, geometrical, constitutional, and functional group descriptors have been selected by GA-PLS to account the antimicrobial activity of the studied compounds. The majority of these descriptors are topological indices. The resulted GA-PLS model possessed very high statistical quality R^2^ = 0.96 and Q^2^ = 0.91. The values of pMIC using PLS model (refined from cross-validation or external prediction set) along with the corresponding relative errors of prediction (REP) are shown in Table 3. Very small values of relative errors confirm the accuracy of the proposed GA-PLS model for modeling antimicrobial activity of the studied compounds.

**Figure 2.**
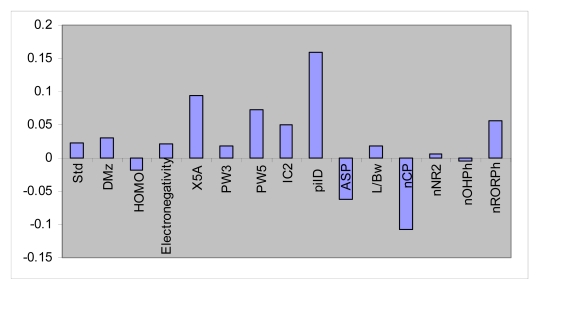
PLS regression coefficients for the variables used in GA-PLS model (against *C. albicans*).

The data set (compounds tested against *C. albicans*, n = 28) was again divided into two groups: calibration set (n = 23) and prediction set (n = 5). Given 23 calibration samples; the leave-one-out cross-validation procedure was used to find the optimum number of latent variables for each PLS model. Here, the most convenient GA-PLS model contained 15 indices, five of them being those obtained by MLR. The PLS estimate of coefficients for these descriptors are given in Figure 2. As it is observed, a combination of quantum, topological, geometrical and functional group descriptors have been selected by GA-PLS to account the antimicrobial activity of the compounds. The majority of these descriptors are topological indices again. The resulted GA-PLS model possessed very high statistical quality R^2^ = 0.91 and Q^2^ = 0.87. The values of pMIC using PLS model along with the corresponding REPs are shown in Table 4. Very small values of relative errors confirm the accuracy of the proposed GA-PLS model for modeling antimicrobial activity of the studied compounds.

### 3.2. FA-MLR and PCRA

Table 5 shows the five factor loadings of the variables (after VARIMAX rotation) for the compounds tested against *S. aureus*. As it is observed, about 79% of variances in the original data matrix could be explained by selected four factors.

Based on the procedure explained in the experimental section, the following three-parametric equation was derived.

(1) $$\begin{array}{c} \text{pMIC}=4.786\;\;\;(\pm 0.484)+\;\;\;0.196(\pm 0.063)\;\;\;\text{DMy}+0.1666\;\;\;(\pm 0.063)\;\;\;\text{nCONHR}-0.130(\pm 0.058)\;\;\;\text{PJ13}\\ {\text{R}}^{\text{2}}=0.73\;\;\;\text{S.}\text{E.}=0.31\;\;\;\text{F}=11.41\;\;\;{\text{Q}}^{\text{2}}=0.68\;\;\;\text{RMScv}=0.34\;\;\;\text{N}=31 \end{array}$$

Equation 1 could explain 73% of the variance and predict 68% of the variance in pMIC data. This equation describes the effect of geometrical (PJI3), functional group (nCONHR) and quantum (DMy) indices on antimicrobial activity.

When factor scores were used as the predictor parameters in a multiple regression equation using forward selection method (PCRA), the following equation was obtained:

(2) $$\begin{array}{c} \text{pMIC}=3.756\;\;\;(\pm 0.036)+\;\;\;0.4000\;\;\;(\pm 0.036)\;\;\;f_{3}\\ {\text{R}}^{\text{2}}=0.81\;\;\;\text{S.}\text{E.}=0.19\;\;\;\text{F}=35.05\;\;\;{\text{Q}}^{\text{2}}=0.79\;\;\;\text{RMScv}=0.20\;\;\;\text{N}=31 \end{array}$$

Equation 2 also shows high equation statistics (81% explained variance and 79% predict variance in pMIC data). Since factor scores are used instead of selected descriptors, and any factor-score contains information from different descriptors, loss of information is thus avoided and the quality of PCRA equation is better than those derived from FA-MLR.

As it is observed from Table 5, in the case of each factor, the loading values for some descriptors are much higher than those of the others. These high values for each factor indicate that this factor contains higher information about which descriptors. It should be noted that all factors have information from all descriptors but the contribution of descriptor in different factors are not equal. For example, factors 1 and 2 have higher loadings for topological, constitutional and functional group indices, whereas information about quantum and functional group descriptors is highly incorporated in factors 3 and 4. Therefore, from the factor scores used by equation E_2_, significance of the original variables for modeling the activity can be obtained. Factor score 1 indicates importance of Mv, HNar, nCaH and IDDE (topological, constitutional and functional group descriptors, respectively). Factor score 2 indicates importance of RBN and Me (constitutional descriptors), Factor score 3 and 4 signify the importance of DMy, and nCONHR (quantum and functional group descriptors, respectively).

Table 6 shows the five factor loadings of the variables (after VARIMAX rotation) for the compounds tested against *C. albicans*. As it is observed, about 80% of variances in the original data matrix could be explained by selected five factors.

Based on the procedure explained in the experimental section, the following four-parametric equation was derived.

(3) $$\begin{array}{c} \text{pMIC}=5.980\;\;\;(\pm 0.695)+0.182\;\;\;(\pm 0.022)\;\;\;\text{piID}-0.167(\pm 0.024)\;\;\;\text{nCp}-0.085(\pm 0.023)\;\;\;\text{ASP}\\ -0.058(\pm 0.023)\;\;\;\text{PW3}\\ {\text{R}}^{\text{2}}=0.81\;\;\;\text{S.}\text{E.}=0.17\;\;\;\text{F}=34.76\;\;\;{\text{Q}}^{\text{2}}=0.79\;\;\;\text{RMScv}=0.18\;\;\;\text{N}=28 \end{array}$$

Equation 3 could explain and predict 85% and 81% of the variance in pMIC data, respectively. This equation describes the effect of topological (piID and PW3), functional group (nCp) and geometrical (ASP) indices on the antimicrobial activity.

When factor scores were used as the predictor parameters in a multiple regression equation using forward selection method (PCRA), the following equation was obtained:

(4) $$\begin{array}{c} \text{pMIC}=3.806(\pm 0.023)+0.237(\pm 0.024)\;\;\;f_{1}-0.114((\pm 0.024)\;\;\;f_{3}+0.081(\pm 0.024)\;\;\;f_{2}-0.065(\pm 0.024)\;\;\;f_{4}\\ {\text{ R}}^{\text{2}}=0.83\;\;\;\text{S.E.}=0.12\;\;\;\text{F}=38.05\;\;\;{\text{Q}}^{\text{2}}=0.81\;\;\;\text{RMScv}=0.12\;\;\;\text{N}=28 \end{array}$$

Equation 4 shows also high equation statistics (88% explained variance and 83% predicted variance in pMIC data). It should be noted that the variables (factor scores) used in Equation 4 are perfectly orthogonal to each other. Since factor scores are used instead of selected descriptors, and any factor-score contains information from different descriptors, loss of information is thus avoided and the quality of PCRA equation is better than those derived from FA-MLR.

As it is observed from Table 6, in the case of each factor, the loading values for some descriptors are much higher than those of the others. Factors 1 and 2 have higher loadings for topological, quantum and functional group indices, whereas information about geometrical, quantum and topological descriptors is highly incorporated in factors 3, 4 and 5. Therefore, from the factor scores used by equation E_4_, significance of the original variables for modeling the activity can be obtained. Factor score 1 indicates importance of PW5, piID and electronegativity (topological and quantum descriptors). Factor score 2 indicates importance of HOMO nCp and nNR_2_ (quantum and functional group descriptor). Factor score 3 signifies the importance of ASP and L/Bw (geometrical descriptors) and factor score 4 and 5 signify the importance of quantum and topological descriptors (DMz and PW3).

Comparison between the results obtained by GA-PLS and the other employed regression methods indicates higher accuracy of this method in describing antimicrobial activity of the studied compounds.

**Figure 3.**
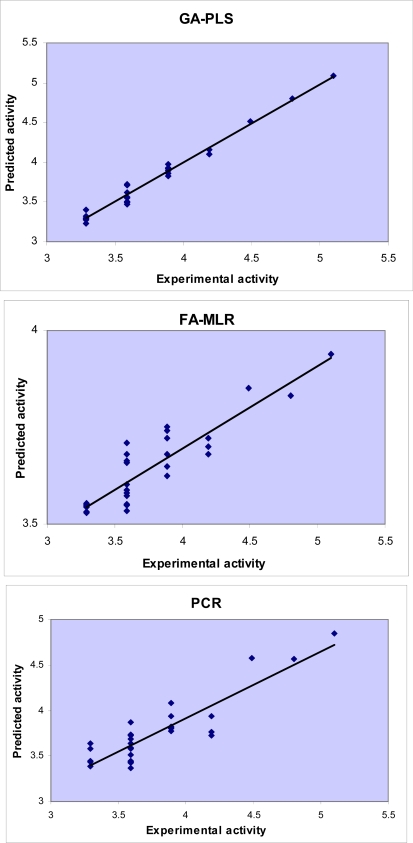
Plots of the cross-validated predicted activity against the experimental activity for the QSAR models obtained by different chemometrics methods (against *S. aureus*).

**Figure 4.**
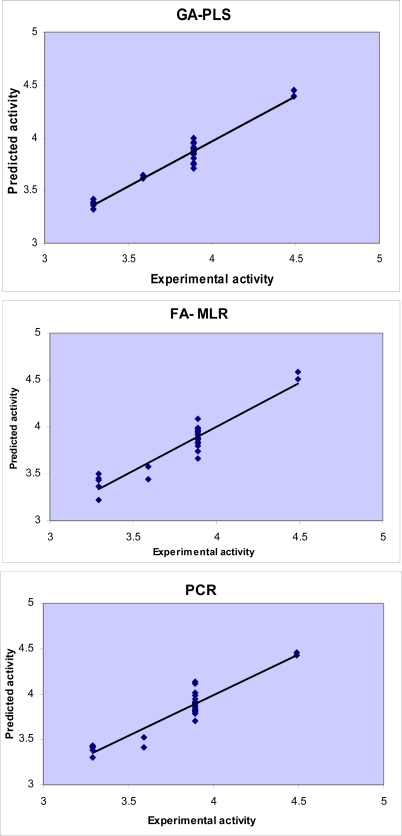
Plots of the cross-validated predicted activity against the experimental activity for the QSAR models obtained by different chemometrics methods (against *C. albicans*).

Difference in accuracy of the different regression methods used in this study is visualized in Figures 3 and 4 by plotting the predicted activity (by cross-validation) against the experimental values. Obviously, all linear models represented scattering of data around a straight line with slope and intercept close to one and zero, respectively. As it is observed, the plot of data resulted by GA-PLS represents the lowest scattering and that obtained by FA-MLR and PCR analysis have lower accuracy. It should be mentioned that the model which GA-PLS method provides is better than that MLR analysis provided in our previous study [25]. In fact, MLR analysis could explain and predict 55% and 35% of variances in the pMIC data (compounds tested against *S. aureus*) and predict 82% and 73% of variances in the pMIC data (compounds tested against *C. albicans*).

## 4. Conclusions

Quantitative relationships between molecular structure and inhibitory activity of a series of 3-hydroxypyridine-4-one and 3-hydroxypyran-4-one derivatives were discovered by a collection of chemometrics methods including GA-PLS, FA-MLR and PCRA. The results revealed the significant role of topological parameters in the antimicrobial activity of the studied compounds against *S. aureus* and *C. albicans*. A comparison between the different statistical methods employed indicated that GA-PLS represented superior results and it could explain and predict 96% and 91% of variances in the pMIC data (compounds tested against *S. aureus*) and predict 91% and 87% of variances in the pMIC data (compounds tested against *C. albicans*). As it is observed, the plot of data resulted by GA-PLS represents the lowest scattering, and the impact of topological descriptors was the most.

## Figures and Tables

**Table 1.** Chemical structure of the compounds used in QSAR analysis. 
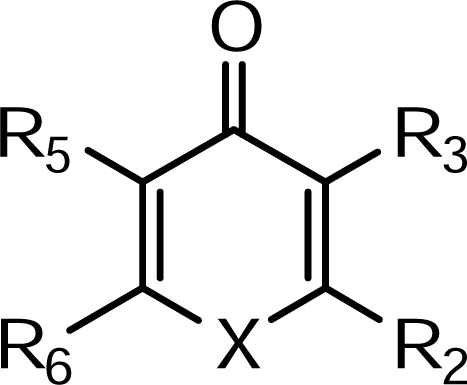

Compound	X	R_2_	R_3_	R_5_	R_6_
**1**	NH	CH_3_	OH	CH_2_-R^a^	H
**2**	NH	C_2_H_5_	OH	CH_2_-R^a^	H
**3**	NH	CH_3_	OH	CH_2_-N(CH_3_)_2_	H
**4**	NH	C_2_H_5_	OH	CH_2_-N(CH_3_)_2_	H
**5**	NH	CH_3_	OH	CH_2_-N(C_2_H_5_)_2_	H
**6**	NH	C_2_H_5_	OH	CH_2_-N(C_2_H_5_)_2_	H
**7**	N-Ph	CH_3_	OH	H	H
**8**	N-*m*-OH-Ph	CH_3_	OH	H	H
**9**	N-C_3_H_7_	CH_3_	OH	H	H
**10**	N-C_4_H_9_	CH_3_	OH	H	H
**11**	O	CH_2_Cl	H	OH	H
**12**	O	CH_3_	H	OH	H
**13**	O	CH_2_OH	OH	H	CH_3_
**14**	O	CH_2_OH	OCH_2_Ph	H	CH_3_
**15**	O	CHO	OCH_2_Ph	H	CH_3_
**16**	O	COOH	OCH_2_Ph	H	CH_3_
**17**	O	CONHR^b^	OCH_2_Ph	H	CH_3_
**18**	O	CONHR^c^	OCH_2_Ph	H	CH_3_
**19**	O	CONHR^d^	OCH_2_Ph	H	CH_3_
**20**	O	CONHR^b^	OH	H	CH_3_
**21**	O	CONHR^c^	OH	H	CH_3_
**22**	O	CONHR^d^	OH	H	CH_3_
**23**	O	CH_2_OH	H	OCH_2_Ph	H
**24**	O	COOH	H	OCH_2_Ph	H
**25**	O	CONHPh	H	OCH_2_Ph	H
**26**	N-CH_3_	CONHPh	H	OCH_2_Ph	H
**27**	N-CH_3_	CONHPh	H	OH	H
**28**	O	CONH-R^e^	H	OCH_2_Ph	H
**29**	N-CH_3_	CONH-R^e^	H	OCH_2_Ph	H
**30**	N-CH_3_	CONH-R^e^	H	OH	H
**31**	O	CH_2_OH	H	OH	H

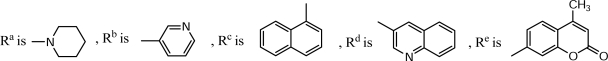

**Table 2.** Brief description of some descriptors used in this study.

Descriptor Type	Molecular Description
Constitutional	Mean atomic van der Waals volume (Mv) (scaled on Carbon atom), no. of heteroatoms, no. of multiple bonds (nBM), no. of rings, no. of circuits, no of H-bond donors, no of H-bond acceptors, no. of Nitrogen atoms (nN), chemical composition, sum of Kier-Hall electrotopological states (Ss), mean atomic polarizability (Mp), number of rotable bonds (RBN), mean atomic Sanderson electronegativity (Me), etc.
Topological	Narumi harmonic topological index (HNar), Total structure connectivity index (Xt), information content index (IC), mean information content on the distance degree equality (IDDE), total walk count, path/walk-Randic shape indices (PW3, PW4, PW5, Zagreb indices, Schultz indices, Balaban J index (such as MSD) Wiener indices, Information content index (neighborhood symmetry of 2-order) (IC2), Ratio of multiple path count to path counts (PCR), Lovasz-Pelikan index (leading eigenvalue) (LP1), total information content index (neighborhood symmetry of 1-order) (TIC1), reciprocal hyper-detour index (Rww), Average connectivity index chi-5 (X5A), piID (conventional bond-order ID number), etc.
Geometrical	3D Petijean shape index (PJI3), Asphericity (ASP), Gravitational index, Balaban index, Wiener index, Length-to-breadth ratio by WHIM (L/Bw), etc.
Quantum	Highest occupied Molecular Orbital Energy (HOMO), Lowest Unoccupied Molecular Orbital Energy (LUMO), Most positive charge (MPC), Sum of square of positive charges (SSPC), Sum of square of negative charges (SSNC), Sum of positive charges (SUMPC), Sum of negative charges (SUMNC), Sum of absolute of charges (SAC), Standard deviation (Std), Total dipole moment (DM_t_), Molecular dipole moment at X-direction (DM_X_), Molecular dipole moment at Y-direction (DM_Y_), Molecular dipole moment at Z-direction (DM_Z_), Electronegativity (χ= −0.5 (HOMO-LUMO)), Electrophilicity (ω= χ^2^/2 η), Hardness (η = 0.5 (HOMO+LUMO)), Softness (S=1/ η).
Functional group	Number of total secondary C(sp3) (nCs), Number of total tertiary carbons (nCt), Number of H-bond acceptor atoms (nHAcc), Number of secondary amides (aliphatic) (nCONHR), Number of unsubstituted aromatic C (nCaH), Number of ethers (aromatic) (nRORPh), Number of ketones (aliphatic) (nCO), Number of tertiary amines (aliphatic) (nNR2), Number of phenols (nOHPh), Number of total primary C(sp3) (nCp), etc.
Chemical	LogP (Octanol-water partition coefficient), Hydration Energy (HE), Polarizability (Pol), Molar refractivity (MR), Molecular volume (V), Molecular surface area (SA).

**Table 3.** Experimental and predicted activity of compounds against *Staphylococcus aureus.*

Compound	Experimental pMIC^a^	Predicted pMIC	REP b (%)
**1**	3.29	3.3205	0.9173
**2**	3.29	3.3007	0.3242
**3**	3.29	3.2266	−1.9664
4*	3.29	3.3976	3.1675
**5**	4.19	3.7498	−11.740
**6**	3.29	3.3205	0.9173
**7**	3.89	3.8255	−1.6850
**8**	3.29	3.2698	−0.6172
**9**	3.29	3.2886	−0.0440
10*	3.89	3.9283	0.9738
**11**	3.59	3.6207	0.8470
**12**	3.59	3.7254	3.6340
**13**	3.59	3.5063	−2.3883
**14**	3.59	3.6212	0.8627
15*	4.19	4.1563	−0.8119
**16**	3.59	3.5611	−0.8123
**17**	3.59	3.6177	0.7647
**18**	3.59	3.5548	−0.9915
19*	3.89	3.8950	0.1293
**20**	4.19	4.0995	−2.2079
**21**	3.59	3.7117	3.2787
**22**	5.10	5.0840	−0.3141
**23**	3.59	3.5533	−1.0318
24*	3.59	3.7223	3.5534
**25**	3.89	3.9222	0.8214
**26**	3.89	3.9779	2.2092
**27**	4.80	4.8022	0.0453
**28**	3.89	3.8591	−0.8011
**29**	3.59	3.4907	−2.8470
30*	4.49	4.5105	0.4549
**31**	3.59	3.4728	−3.3746

^a^ pMIC= −log (MIC),

^b^ REP = Relative Error Prediction

*Compounds used as prediction set

**Table 4.** Experimental and predicted activity of compounds against *Candida albicans.*

Compd.	Experimental pMIC	Predicted pMIC	REP(%)
**2**	3.29	3.4139	3.6304
4*	3.29	3.3893	2.9303
**5**	3.89	3.8920	0.0514
**6**	3.29	3.3591	2.0577
**7**	3.29	3.3835	2.7631
**8**	3.59	3.6477	1.5813
**9**	3.29	3.3208	0.9272
10*	3.59	3.6196	0.8175
**11**	3.89	3.9567	1.6857
**12**	3.89	3.7481	−3.7870
**13**	3.89	3.9092	0.4922
**14**	3.89	3.7076	−4.9191
**15**	3.89	3.8892	−0.0203
**16**	3.89	3.8422	−1.2433
17*	4.49	4.3961	−2.1360
**18**	4.49	4.4476	−0.9524
**19**	3.89	3.7076	−4.9191
**20**	3.89	3.8014	−2.3296
**21**	3.89	3.9525	1.5813
**23**	3.89	3.7450	−3.8727
24*	3.89	3.9056	0.3994
**25**	3.89	3.9969	2.6755
**26**	3.89	3.8489	−1.0691
**27**	3.89	3.7573	−3.5304
**28**	3.89	3.9503	1.5262
29*	3.89	3.9964	2.6619
**30**	3.89	3.8978	0.2006
**31**	3.89	3.8732	−0.4333

*Compounds used as prediction set

**Table 5.** Numerical values of factor loading numbers 1–4 for some descriptors after VARIMAX rotation (against *S. aureus*).

	1	2	3	4	Commonality
**MPC**	0.588	−0.105	0.587	−0.313	0.799
**DMy**	0.195	−0.054	0.762	0.071	0.627
**HOMO**	0.059	0.637	−0.013	0.620	0.794
**Electonegativity**	−0.643	−0.206	−0.199	−0.496	0.741
**Mv**	0.751	−0.413	0.362	−0.259	0.934
**Me**	0.001	−0.781	0.097	−0.298	0.708
**RBN**	0.087	0.902	0.068	0.003	0.826
**HNar**	0.866	0.051	0.217	−0.252	0.863
**Xt**	−0.645	−0.505	−0.307	0.081	0.772
**IDDE**	0.746	0.359	0.215	0.324	0.837
**LP1**	0.667	0.460	0.368	0.292	0.877
**TIC1**	0.714	0.413	0.175	0.127	0.726
**PJI3**	0.375	0.611	−0.315	−0.276	0.689
**nCS**	−0.559	0.578	−0.411	0.199	0.855
**nCaH**	0.894	−0.140	−0.143	−0.079	0.845
**nCONHR**	0.261	0.220	0.695	−0.906	0.765
**nCO**	−0.082	0.081	−0.214	0.853	0.787
**pMIC*S. aureus***	0.041	−0.116	0.898	−0.051	0.824
**%variance**	29.87	20.10	17.15	12.12	79.24

**Table 6.** Numerical values of factor loading numbers 1–5 for some descriptors after VARIMAX rotation (against *C. albicans*).

	1	2	3	4	5	Commonality
**Std**	−0.491	−0.431	−0.459	−0.107	0.095	0.657
**DMz**	−0.007	0.102	−0.209	0.860	0.322	0.898
**HOMO**	0.240	0.811	−0.156	−0.349	0.014	0.861
**Electonegativity**	−0.706	−0.389	0.142	0.323	−0.310	0.871
**X5A**	−0.627	−0.664	−0.134	−0.102	0.129	0.879
**PW3**	−0.166	0.594	−0.377	−0.158	0.893	0.584
**PW5**	0.913	−0.079	0.055	0.135	−0.132	0.879
**IC2**	0.579	0.272	−0.164	0.210	0.584	0.820
**piID**	0.750	−0.070	−0.333	−0.190	−0.208	0.758
**ASP**	−0.075	0.087	0.866	−0.198	0.322	0.905
**L/Bw**	0.064	0.117	0.926	−0.023	0.164	0.902
**nCp**	−0.206	0.754	−0.224	−0.097	−0.325	0.777
**nNR2**	−0.366	0.722	0.148	0.287	−0.234	0.814
**nOHPh**	−0.191	−0.415	−0.165	−0.447	0.356	0.562
**nRORPh**	0.571	−0.522	0.379	0.002	−0.341	0.858
**pMIC*C. albicans***	0.628	−0.627	−0.277	−0.107	0.602	0.872
**%variance**	22.58	20.58	14.71	14.02	8.71	80.60

## References

[1] Schmidi H (1997). Multivariate Prediction for QSAR. Chemom. Intell. Lab. Sys.

[2] Hansch C, Kurup A, Garg R, Gao H (2001). Chem-bioinformatics and QSAR. A Review of QSAR Lacking Positive Hydrophobic Terms. Chem. Rev.

[3] Wold S, Trygg J, Berglund A, Antii H (2001). Some Recent Developments in the PLS Modeling. Chemom. Intell. Lab. Syst.

[4] Hemmateenejad B, Miri R, Akhond M, Shamsipur M (2002). QSAR Study of the Calcium Channel Antagonist Activity of some Recently Synthesized Dihydropyridine Derivatives. An Application of Genetic Algorithm for Variable Selection in MLR and PLS Methods. Chemom. Intell. Lab. Syst.

[5] Hemmateenejad B, Miri R, Akhond M, Shamsipur M (2002). Quantitative Structure Activity Relationship Study of Recently Synthesized 1,4- Dihydropyridine Calcium Channel Antagonists. Application of Hansch Analysis Methods. Arch. Pharm. Pharm. Med. Chem.

[6] Horvath D, Mao B (2003). Neighborhood Behavior. Fuzzy Molecular Descriptors and Their Influence on the Relationship between Structural Similarity and Property Similarity. QSAR. Comb. Sci.

[7] Putta S, Eksterowicz J, Lemmen C, Stanton R (2003). A Novel Subshape Molecular Descriptor. J. Chem. Inf. Comput. Sci.

[8] Gupta S, Singh M, Madan AK (1999). Superpendentic Index: A Novel Topological Descriptor for Predicting Biological Activity. J. Chem. Inf. Comput. Sci.

[9] Consonni V, Todeschini R, Pavan M (2002). Structure/Response Correlations and Similarity/Diversity Analysis by GETAWAY Descriptors. 2. Application of the Novel 3D Molecular Descriptors to QSAR/QSPR Studies. J. Chem. Inf. Comput. Sci.

[10] Deeb O, Hemmateenejad B, Jaber A, Garduno-Juarez R, Miri R (2007). Effects of the Electronic and Physicochemical Parameters on the Carcinogenecis Activity of Some Sulfa Drug Using QSAR Analysis Based on Genetic-MLR & Genetic-PLS. Chemosphere.

[11] Eaton JW, Brandt P, Mahoney JR, Lee JT (1982). Haptoglobin: A Natural Bacteriostat. Science.

[12] Jones RL, Peterson CM, Grady RW, Kumbaraci T, Cerami A (1977). Effects of Iron Chelators and Iron Overload on *Salmonella* Infection. Nature.

[13] Weinberg ED (1990). Cellular Iron Metabolism in Health and Diseased. Drug Metab. Rev.

[14] Weinberg ED (1978). Iron and Infection. Microbiol. Rev.

[15] Weinberg ED (1984). Iron Withholding: A Defense Against Infection and Neoplasia. Physiol. Rev.

[16] Skaar EP, Gaspar AH, Schneewind O (2006). *Bacillus anthracis* IsdG, a Heme-Degrading Monooxygenase. J. Bacteriol.

[17] Neilands JB (1995). Siderophores: Structure and Function of Microbial Iron Transport Compounds. J. Biol. Chem.

[18] Sebat JL, Paszczynski AJ, Cortese MS, Crawford RL (2001). Antimicrobial Properties of Pyridine-2,6-Dithiocarboxylic Acid, a Metal Chelator Produced by *Pseudomonas spp*. Appl. Envir. Microbiol.

[19] Weinberg GA (1994). Iron Chelators as Therapeutic Agents against *Pneumocystis carinii. Antimicrob*. Agents Chemother.

[20] van Asbeck BS, Marcelis JH, Marx JJM, Struyvenberg A, van Kats JH, Verhoef J (1983). Inhibition of Bacterial Multiplication by the Iron Chelator Deferoxamine: Potentiating Effect of Ascorbic Acid. Eur. J. Clin. Microbiol. Infect. Dis.

[21] Erol DD, Yulug N (1994). Synthesis and Antimicrobial Investigation of Thiazolinoalkyl-4(1*H*)-pyridones. Eur. J. Med. Chem.

[22] Min-Hua F, van der Does L, Bantjes A (1993). Iron (III)-Chelating Resins. 3. Synthesis, Iron (III)-Chelating Properties, and *in vitro* Antibacterial Activity of Compounds Containing 3-hydroxy-2-methyl-4(1H)-pyridinone Ligands. J. Med. Chem.

[23] Aytemir MD, Erol DD, Hider RC, Ozalp M (2003). Synthesis and Evaluation of Antimicrobial Activity of New 3-Hydroxy-6-methyl-4-oxo-4*H*-pyran-2- carboxamide Derivatives. Turk. J. Chem.

[24] Aytemir MD, Hider RC, Erol DD, Ozalp M, Ekizoglu M (2003). Synthesis of New Antimicrobial Agents; Amide Derivatives of Pyranones and Pyridinones. Turk. J. Chem.

[25] Fassihi A, Abedi D, Saghaie L, Sabet R, Fazeli H, Bostaki Gh, Deilami O, Sadinpour H (2008). Synthesis, Antimicrobial Evaluation and QSAR Study of Some 3-hydroxypyridine-4- one and 3-hydroxypyran-4-one Derivatives. Eur. J. Med. Chem.

[26] Todeschini R http://michem.disat.unimib.it/.

[27] Frisch MJ, Trucks MJ, Schlegel HB, Scuseria GE, Robb MA, Cheeseman JR, Zakrzewski VG, Montgomery JA, Stratmann JR, Burant JC (1998). Gaussian 98, Revision A.7.

[28] Roy K (2003). QSAR of Adenosine Receptor Antagonists II: Exploring Physicochemical Requirements for Selective Binding of 2-arylpyrazolo [3,4-c] quinoline Derivatives with Adenosine A^1^ and A^3^ Receptor Subtypes. QSAR. Comb. Sci.

[29] Siedlecki W, Sklansky J (1988). On Automatic Feature Selection. Int. J. Pattern Recog. Artif. Intell.

[30] Leardi R (2000). Application of Genetic Algorithm-PLS for Feature Selection in Spectral Data Sets. J. Chemomtr.

[31] Leardi R, Gonzalez AL (1998). Genetic Algorithm Applied to Feature Selection in PLS Regression: How and When to Use Them. Chemom. Intell. Lab. Syst.

[32] Fassihi A, Sabet R (2008). QSAR Study of p56^lck^ Protein Tyrosine Kinase Inhibitory Activity of Flavonoid Derivatives Using MLR and GA-PLS. Int. J. Mol. Sci.

[33] Leardi R (2001). Genetic Algorithms in Chemometrics and Chemistry: A Review. J. Chemometrics.

[34] Hemmateenejad B (2004). Optimal QSAR Analysis of the Carcinogenic Activity of Drugs by Correlation Ranking and Genetic Algorithm-Based. J. Chemometrics.

[35] Franke R, Gruska A, van Waterbeemd H (1995). Chemometrics Methods in Molecular Design. Methods and Principles in Medicinal Chemistry.

[36] Kubinyi H, Wolff ME (1995). The Quantitative Analysis of Structure-Activity Relationships. Burger’s Medicinal Chemistry and Drug Discovery.

